# Temporal Relationship Between Seasonal Burn Incidence and Clinical Severity in a Long-Term Regional Cohort

**DOI:** 10.7759/cureus.101595

**Published:** 2026-01-15

**Authors:** Saleh Alhotan, Abdulaziz Aljasser, Shoug Alaodah, Mona Almansour, Renad Alrish, Rawan Alkhalaf, Mansour Alharbi, Abdullah Alzain, Bassem Alomari

**Affiliations:** 1 Department of Surgery, College of Medicine, Qassim University, Buraidah, SAU; 2 Department of Surgery, Qassim University Medical City, Buraidah, SAU; 3 Plastic Surgery, King Fahad Specialist Hospital, Buraidah, SAU; 4 Emergency Medicine, King Fahad Specialist Hospital, Buraidah, SAU; 5 Adult Critical Care Nursing, Burn Unit, King Fahad Specialist Hospital, Buraidah, SAU; 6 Nursing, King Fahad Specialist Hospital, Buraidah, SAU; 7 College of Medicine, Qassim University, Buraidah, SAU; 8 College of Medicine, Sulaiman Alrajhi University, Buraidah, SAU

**Keywords:** burn, epidemiology, injury, plastic surgery, seasonal changes

## Abstract

Background: Seasonal changes have a measurable impact on burn frequency and clinical implications, yet the relationship with injury severity remains understudied in arid regions. This study evaluated the association between seasonal and cultural burn trends in the Qassim region, focusing on the dissociation between admission volume and injury severity.

Methods: A retrospective registry analysis was conducted for all acute burn admissions to the regional referral center from November 2018 to November 2025 (n=816). Monthly admission volume was examined using negative binomial regression, and injury severity, measured as percent total body surface area burned (%TBSA), was analyzed using RStudio (Posit, Boston, MA). A two-sided p-value < 0.05 was deemed statistically significant. Data were categorized by age, season, and cultural time (Ramadan/Eid).

Results: Injury severity varied significantly by season (p = 0.012). For instance, winter admissions had a higher median TBSA (5%, interquartile range (IQR) 0.3-15%) than summer admissions (median TBSA = 1%; IQR 0.1-13.5%). In contrast, the aggregate admission volume did not differ considerably by season. Pediatric admissions (<15 years, n=322; 39.5%) had a consistent distribution throughout the year, but adult admissions (n=494; 60.5%) had a descriptive spring peak that did not achieve statistical significance after adjustment. Scald injuries (n=359; 44.0%) were the most common mechanism across all seasons, while winter-specific analysis revealed no significant TBSA difference between scald and flame injuries. Cultural periods exhibited predictable temporal clustering but no statistically significant increase in volume.

Conclusion: Burn epidemiology in Qassim is characterized by a stable year-round frequency driven by pediatric injuries, while dissociated from a marked winter increase in injury severity in adults. Prevention strategies should prioritize year-round pediatric safety alongside focused interventions to mitigate severe winter-associated domestic risks.

## Introduction

Burn injuries are a well-established global public health concern, ranking among the main causes of trauma-related morbidity and mortality worldwide [1,2]. According to the Global Burden of Diseases, approximately nine million new burn injuries required medical attention in 2019 and resulted in over 120,000 fatalities, the majority of which occurred in low- and middle-income countries (LMICs) with limited acute care capacity [1,2]. Over 90% of fatal fire‑related burns occur in LMICs, and South‑East Asia alone accounts for more than half of these deaths [2].

Seasonal changes are a major modulator of burn epidemiology. Hence, retrospective analyses from Portugal, Turkey, North Carolina, and Switzerland demonstrate that burn admissions tend to cluster in the winter months, when indoor heating, open flame devices, and combustible fuels increase the risk of fire-related injuries [3-6]. In contrast, spring and summer peaks have been reported in Bangladesh, India, and Sub‑Saharan Africa, where outdoor cooking, agricultural work, and the preparation of traditional foods increase exposure to hot liquids and flames [2]. Notably, seasonality may alter both the frequency and severity of burns. A recent study from Turkey found that the number of people admitted to the hospital was varied across seasons and the severity of their injuries, assessed by TBSA (%), was different. There were different patterns in the summer and winter months [4].

Cultural periods significantly influence burn risk, particularly in the Middle East [7]. In Muslim regions, Ramadan is associated with altered cooking patterns, increased nighttime meal preparation, and fatigue-related lapses in supervision [2]. These behavioral shifts, especially among women responsible for domestic activities, have been linked to a rise in scald and flame injuries [8-10]. Similarly, the Eid al-Fitr and Eid al-Adha festivals contribute to short-term increases in thermal injury risk through large-scale festive cooking, outdoor grilling, and the use of fireworks [3,11].

More importantly, Children bear a disproportionate share of this burden; burns are the sixth most common cause of death among 5- to 14‑year‑old children and the fourth-ranked injury globally that requires medical attention [12-15]. Among pediatric populations, burn incidence peaks in children under five years of age, a group that depends entirely on caregivers and lacks self‑protection abilities [2,12]. According to studies from Ghana, Iran, the United Arab Emirates (UAE), and India, infants and toddlers (0-4 years) account for up to 50% of all pediatric burn hospitalizations, with scald injuries being the most common [2,16,17]. In Saudi Arabia, a systematic review conducted on 3,308 patients found that 52% of burn incidences occurred in children under the age of 10 years, and scalding accounted for 62% of injuries [18]. These patterns highlight the urgent need for age‑targeted prevention strategies.

Despite Saudi Arabia's tremendous socioeconomic progress, burn-related morbidity remains a significant concern. Local hospital-based studies from Riyadh, Jeddah, and Dammam show that 83% of burns occur at home, with males outnumbering females substantially [18,19]. However, most published papers are single-site, cross-sectional, or have short observation periods, making it impossible to conduct a thorough examination of temporal trends [18]. The Qassim region, characterized by a desert climate with extreme summer heat and cold winters, lacks longitudinal data on seasonal and cultural injury clustering. This study aims to evaluate a seven-year dataset from the region's central referral center. To decipher if burn admission volume and injury severity in Qassim have a seasonal pattern, as well as how cultural periods (Ramadan and Eid) and patient age affect these trends.

## Materials and methods

Study design and setting

This study was a retrospective observational analysis conducted at King Fahad Specialist Hospital (KFSH) in Buraidah. KFSH is the principal burn referral facility for the Qassim Health Cluster, receiving patients from across the region. The study followed the Strengthening the Reporting of Observational Studies in Epidemiology (STROBE) guidelines [20] (see supplementary 1 in Appendices). The study utilized a seven-year dataset covering all acute burn presentations from November 1, 2018, to November 30, 2025. All patients admitted with acute thermal, chemical, or electrical burn injuries were eligible for inclusion. Exclusion criteria included friction injuries without tissue loss, chronic non-burn wounds, and readmissions for the same injury episode.

Variables and definitions

Temporal Variables

Seasonality was defined meteorologically: Winter (December-February), Spring (March-May), Summer (June-August), and Autumn (September-November). Cultural periods included Ramadan, which was coded using the full lunar-month date range for each Gregorian year and the Eid holidays (Eid al-Fitr and Eid al-Adha), which were captured using a three- to four-day window surrounding the holiday dates to account for pre-celebration preparations and festivities (see supplementary 2, 3, and 4 in Appendices).

Clinical Variables

Data points included age, sex, mechanism of injury, and Total Body Surface Area (TBSA). To identify age-related risk factors, the population was divided into pediatric (<15 years) and adult (≥15 years) subgroups.

Outcomes

The primary outcomes were monthly admission volume and injury severity. Injury severity was assessed using burn size, measured as the percentage of total body surface area burned (TBSA, %). This seasonal analysis did not focus on mortality and length of stay, which were recorded in the registry.

Data handling

Baseline demographic and clinical data were extracted for the full cohort of 816 patients. Admission dates were complete for all cases. TBSA data was available for 806 patients (98.8%), and age was available for all 816 patients (100%). Missing data for other variables was negligible (<1%) and handled through complete-case analysis.

Statistical analysis

Categorical variables were summarized as counts and percentages, while continuous variables (TBSA, age) were reported as medians with interquartile ranges (IQR) due to non-normal distribution. (a) Group comparisons: burn mechanism distributions were compared across seasons using the chi-square test. Seasonal differences in injury severity, assessed by TBSA (%), were evaluated using the Kruskal-Wallis rank-sum test. Sex-based differences in TBSA during the winter season were evaluated using the Wilcoxon rank-sum test. Fisher’s exact test was applied to evaluate the association between the mechanism of injury and the defined cultural periods. (b) Time-series analysis: monthly case counts were analyzed using negative binomial regression adjusted for a calendar year to control for long-term population trends. Incidence rate ratios (IRRs) with 95% confidence intervals (CIs) were calculated. (c) Model selection: a Poisson regression model was initially used. Tests for overdispersion revealed a dispersion ratio of 1.14, indicating an acceptable match. However, a negative binomial model was used to ensure resilience against modest deviations from Poisson assumptions as well as conformity with normal burn epidemiology methodologies. All analyses were performed using RStudio (Posit, Boston, MA). A two-sided p-value < 0.05 was considered statistically significant.

Ethical approval

The study received formal approval from the Qassim Regional Research Ethics Committee (Registration No. 607-47-5877). Approval was granted under the expedited review category for implementation. The committee waived the requirement for informed consent because the study used retrospective, fully anonymized data with no direct patient contact.

## Results

Baseline characteristics

A total of 816 patients were admitted during the seven-year study period (November 2018 to November 2025). The cohort was predominantly male (n = 573; 70.2%), with a male-to-female ratio of 2.4:1. The mean age was 24.1 years (SD 20.5), and the median age was 24 years (IQR 3-37), with a range from neonates (one day) to 94 years (Table 1).

**Table 1:** Baseline characteristics of the study population (n = 816) TBSA: Total body surface area.

Variables	Values
Total patients	816
Age (n=816):	
Mean age	24.1 years (SD 20.5)
Median age	24 years (IQR 3-37)
Range	1 day - 94 years
Sex:	
Male	573 (70.2%)
Female	243 (29.8%)
Mechanism of injury (n=816)	
Scald	359 (44.0%)
Flame	275 (33.7%)
Electrical	62 (7.6%)
Chemical	50 (6.1%)
Contact	25 (3.1%)
Friction	23 (2.8%)
Inhalation injury	2 (0.2%)
Sunburn	1 (0.1%)
Other	18 (2.2%)
Missing	1 (0.1%)
TBSA (n=806)	
Median	5% (IQR 0.14-11%)
Mean	8.7% (SD 13.1%)
Range	0-99%

Seasonal distribution and regression analysis

**Figure 1:**
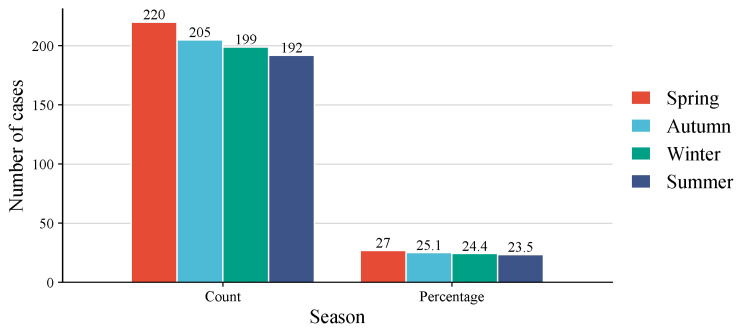
Seasonal distribution of acute burn admissions (n = 816) The bar chart illustrates total admission counts across the four meteorological seasons from November 2018 to November 2025. Percentages above the bars represent the proportion of total annual admissions. Negative binomial regression revealed no statistically significant seasonal variation in aggregate admission volume (p = 0.55), highlighting a stable year-round frequency of burn injuries in the Qassim region.

Descriptive analysis showed that Spring accounted for the highest proportion of admissions, followed by Autumn. However, a negative binomial regression, adjusted for calendar year, showed that these differences were not statistically significant. Summer (IRR 0.86, p = 0.10), Autumn (IRR 0.92, p = 0.35), and Winter (IRR 0.91, p = 0.28) did not indicate a significant difference in monthly case volume from Spring (reference). The regression for the calendar year was also non-significant (p = 0.97), indicating that admission volume remained stable during a seven-year period (Table 2 and Figure 1).

**Table 2:** Number and percentage of burn admissions across the four seasons (n = 816) Negative binomial regression adjusted for calendar year did not show a statistically significant association between season and monthly burn incidence (all seasonal incidence rate ratios (IRRs) p > 0.05). Chi-square goodness-of-fit test comparing seasonal distribution of burn admissions: p = 0.55 (not significant).

Season	Number of cases
Spring	220 (27.0%)
Autumn	205 (25.1%)
Winter	199 (24.4%)
Summer	192 (23.5%)

Age-specific seasonal patterns

**Figure 2:**
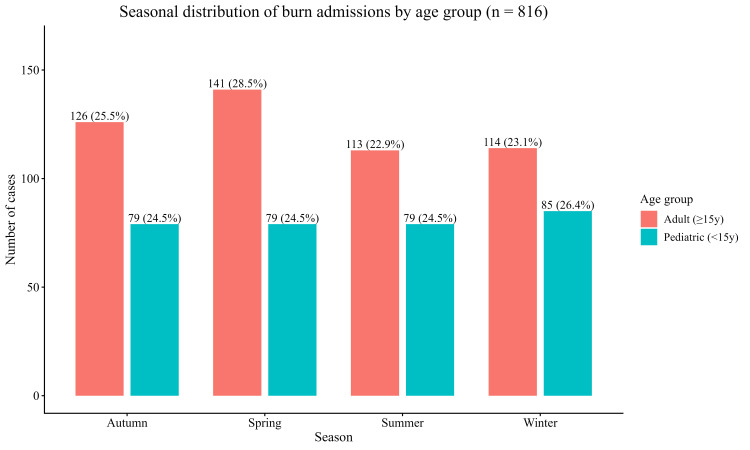
Seasonal distribution of burn admissions stratified by age group Bars show the number of pediatric (<15 years) and adult (≥15 years) admissions across the four seasons (n = 816). Pediatric admissions are relatively evenly distributed, whereas adult cases show a peak in Spring.

Stratification by age group revealed that seasonal variance was mostly driven by adults. Pediatric admissions (<15 years, n = 322; 39.5%) were nearly similarly distributed over the year, where adult admissions (≥15 years, n = 494; 60.5%) showed evident seasonal variability, with a peak in Spring (n=141; 28.5%) and a relative nadir in Summer (n=113; 22.9%) (Figure 2).

Mechanism and TBSA%

**Figure 3:**
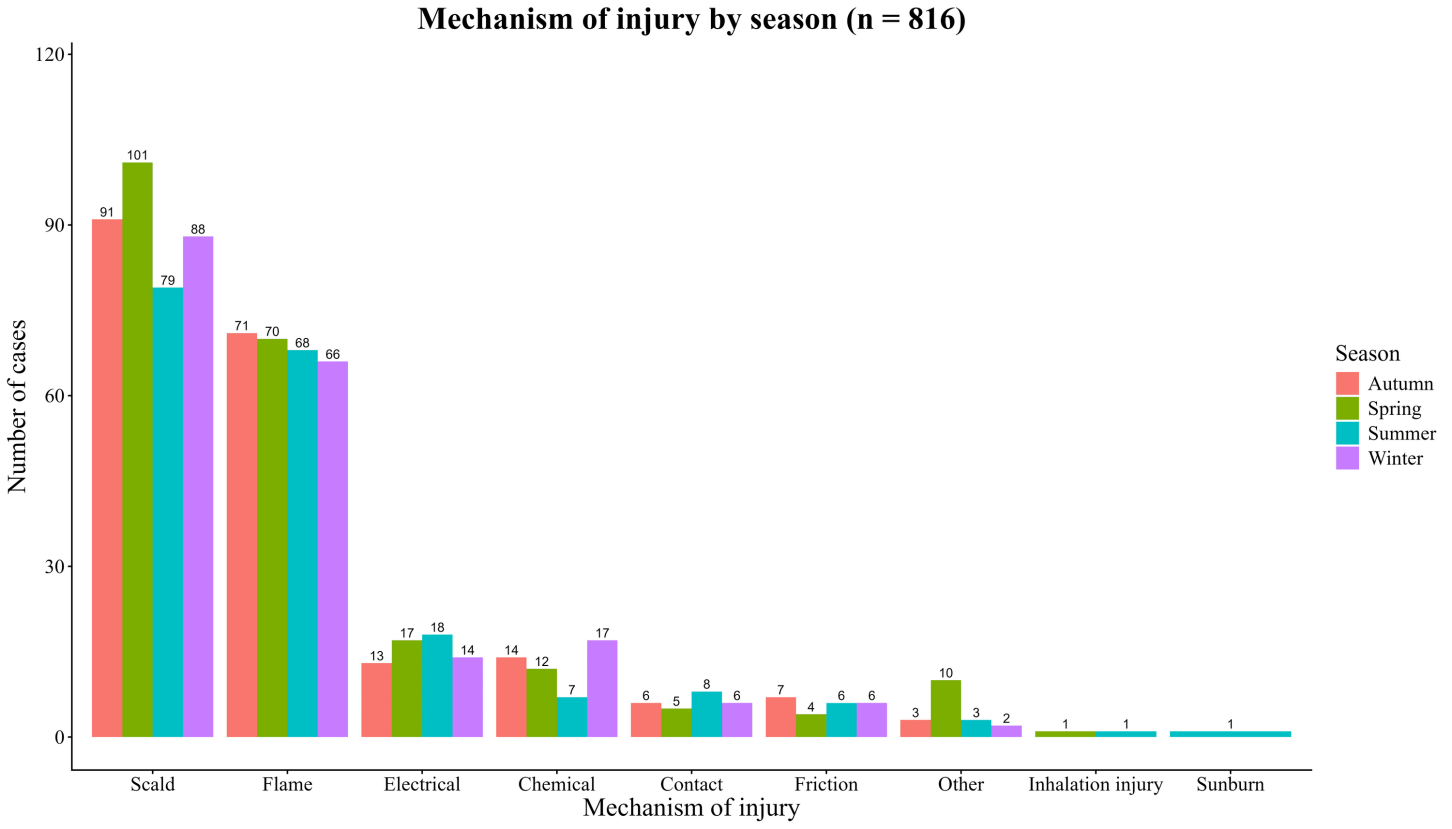
Mechanism of injury by season (n = 816) Stacked bar chart showing the distribution of burn mechanisms (scald, flame, electrical, chemical, contact, friction, etc.) within each of the four seasons.

Scald injuries were the most common mechanism in all seasons, followed by flame burns. Electrical, chemical, contact, and friction injuries were consistently less common, whereas inhalation injuries and sunburn were infrequent. The overall distribution of burn mechanisms did not change considerably over seasons (Table 3 and Figure 3).

**Table 3:** Mechanism distribution across seasons in percentage Chi-square test for association between mechanism of injury and season: χ² = 20.5, df = 24, p = 0.67 (not significant).

	Number of cases per Season in percent			
Causes	Autumn	Spring	Summer	Winter
Scald	44.4%	45.9%	41.0%	44.2%
Flame	34.6%	31.8%	35.6%	33.2%
Electrical	6.3%	7.7%	9.4%	7.0%
Chemical	6.8%	5.5%	3.7%	8.5%
Contact	2.9%	2.3%	4.2%	3.0%
Friction	3.4%	1.8%	3.1%	3.0%
Inhalation injury	0.0%	0.5%	0.5%	0.0%
Other	1.5%	4.5%	1.6%	1.0%
sunburn	0.0%	0.0%	0.5%	0.0%

**Figure 4:**
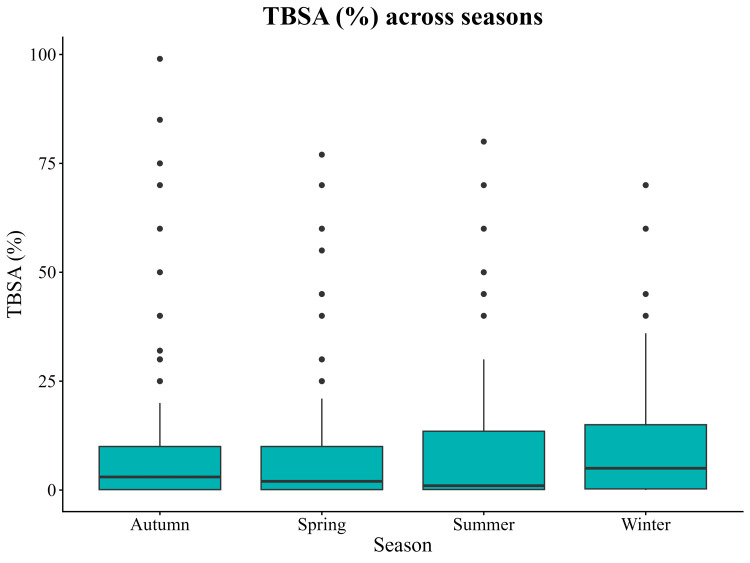
Distribution of burn Total Body Surface Area (TBSA) across seasons (n = 806). Boxplots show the median, interquartile range, and whiskers representing 1.5×IQR. Winter admissions demonstrated the highest injury severity (median TBSA 5%, IQR 0.3-15%), while Summer showed the lowest (median TBSA 1%, IQR 0.1-13.5%). A Kruskal–Wallis test identified a statistically significant difference in TBSA across seasons (χ² = 10.88, df = 3, p = 0.012).

**Figure 5:**
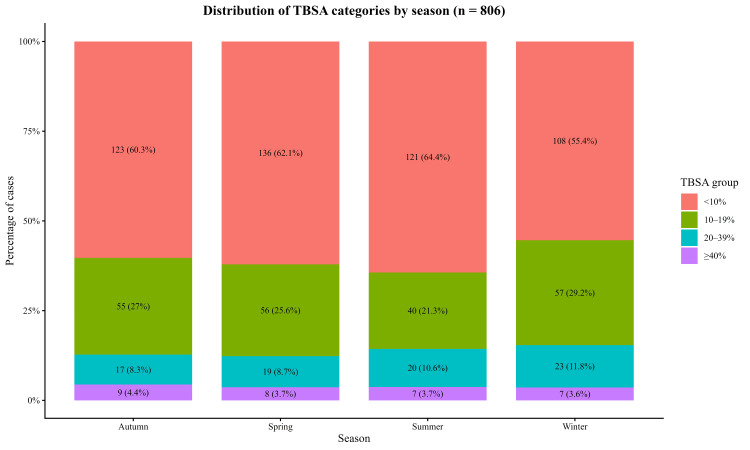
Distribution of burn extent categories (TBSA%) across seasons (n = 806) Stacked bar charts display the proportion and absolute number of cases within TBSA groups (<10%, 10-19%, 20-39%, ≥40%) for each season. Values are shown as n (%) within season. Mild burns (<10% TBSA) predominated across all seasons, while winter demonstrated a higher relative proportion of burns involving ≥10% TBSA, particularly within the 10–19% and 20–39% categories.

Injury severity, measured by TBSA %, demonstrated statistically significant seasonal variation (Kruskal-Wallis χ² = 10.88, df = 3, p = 0.012). Complete TBSA data were available for 806 cases. Winter admissions showed the highest burn severity. In contrast, summer admissions had the lowest severity. Spring and Autumn presented intermediate patterns. (Table 4 and Figures 4, 5).

**Table 4:** Burn severity (TBSA %) across seasons Kruskal-Wallis test comparing TBSA across seasons: χ² = 10.88, df = 3, p = 0.012.

Season	No. of cases	Median	IQR	Mean	SD
Autumn	204	3%	0.1-10%	8.65%	14.40%
Spring	219	2%	0.1-10%	8.01%	12.40%
Summer	188	1%	0.1-13.5%	8.14%	12.80%
Winter	195	5%	0.3-15%	9.91%	12.90%

Sex-stratified winter analysis

**Figure 6:**
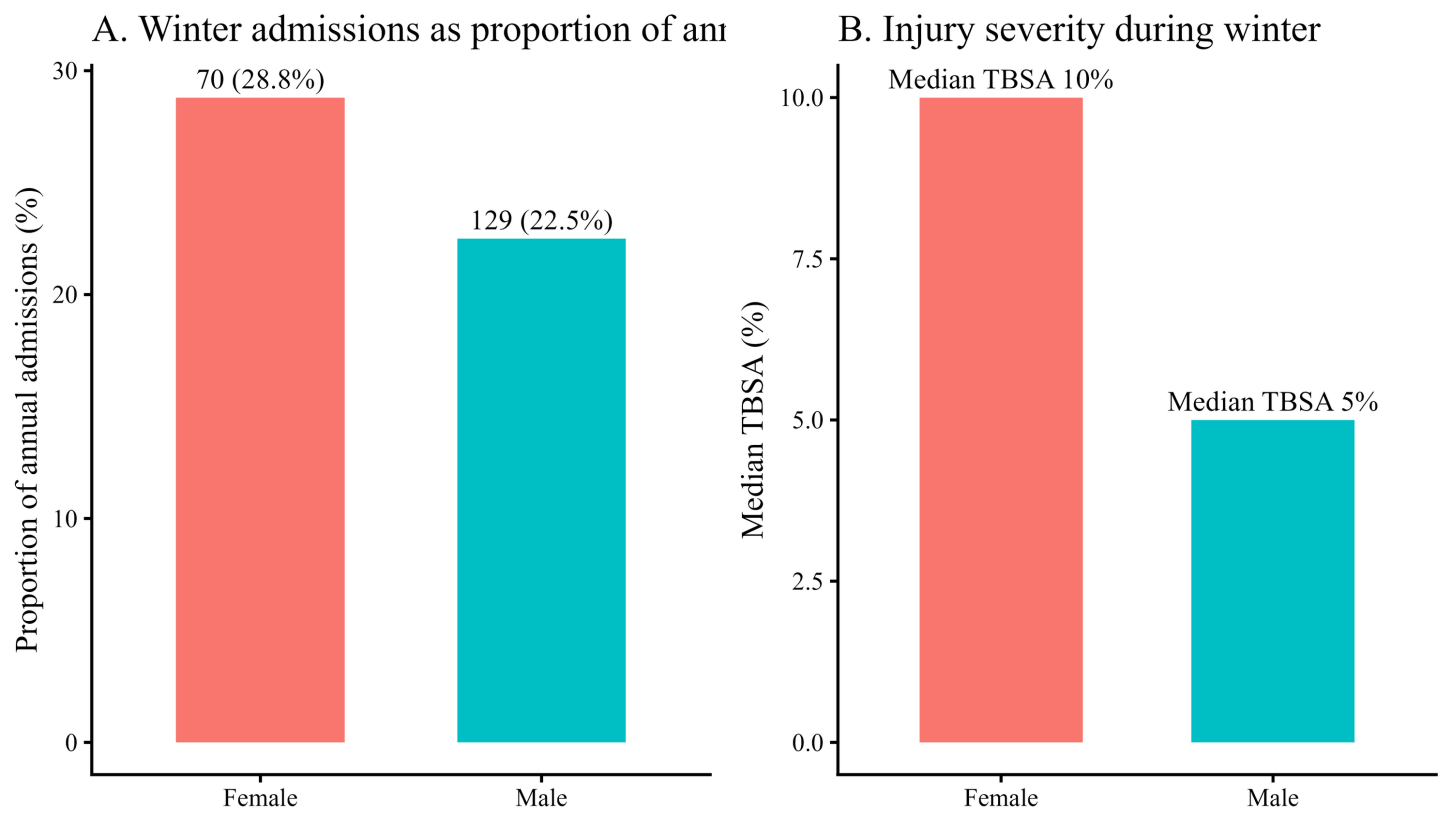
Sex-stratified analysis of winter burn admissions Panel A shows winter admissions expressed as a proportion of each sex’s total annual admissions, with females accounting for 70 (28.8%) cases and males for 129 (22.5%). Panel B illustrates median injury severity during winter, measured by total body surface area (TBSA), which was higher among females (median 10%) than males (median 5%), although this difference did not reach statistical significance (Wilcoxon rank-sum test, p = 0.17).

During winter, males accounted for a higher absolute number of admissions (129) than females (70). However, when adjusted for annual admission volume, winter admissions accounted for a greater proportion of total cases among females (70, 28.8%) than males (129, 22.5%). Among patients with available TBSA data, the median burn size in winter was higher in females (10%) than in males (5%), although this difference did not reach statistical significance (Wilcoxon rank-sum test, p = 0.17) (Figure 6).

Cultural periods

**Figure 7:**
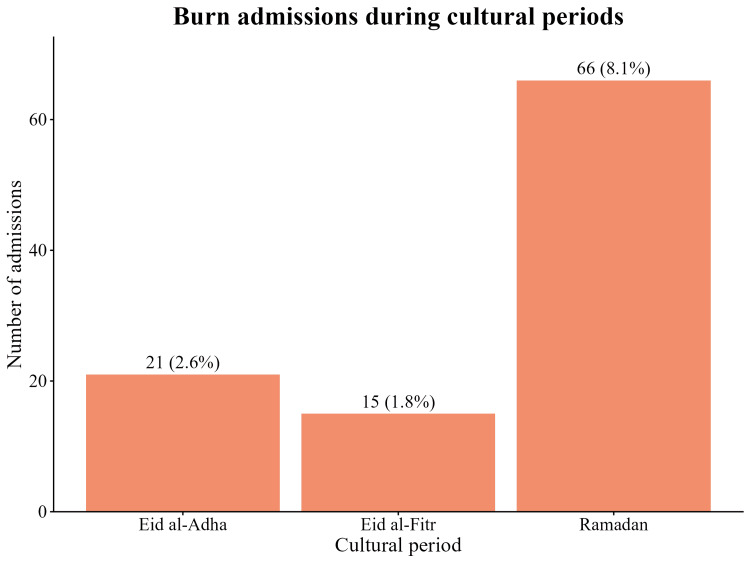
Burn admissions during cultural periods Number and percentage of admissions occurring during Ramadan (66 cases; 8.1%), Eid al-Fitr (15 cases; 1.8%), and Eid al-Adha (21 cases; 2.6%) across the seven-year study period (n = 816). These cultural clusters constitute a small proportion of total admissions but reflect identifiable temporal patterns aligned with domestic and holiday activities.

A total of 66 admissions occurred during Ramadan, accounting for 8.1% of all cases. This period showed an injury pattern aligned with household and domestic activity. Additional clusters were observed during the Eid holidays: 15 admissions (1.8%) occurred during the Eid al-Fitr window, and 21 admissions (2.6%) occurred during the Eid al-Adha window. The distribution of burn mechanisms during Ramadan did not differ significantly from non-Ramadan periods (Fisher’s exact test, p = 0.27). Scald and flame injuries accounted for the majority of cases during both Ramadan and non-Ramadan periods (Figure 7).

## Discussion

Although seasonal burn patterns are widely recognized, the distinction between seasonal frequency and injury severity is often overlooked, particularly in arid regions. Elucidating whether seasonal risks drive exposure volume or injury intensity is vital for refining public health interventions and understanding the specific epidemiological nuances of the Qassim region. This seven-year retrospective analysis is the most thorough assessment of temporal burn patterns in the Qassim region to date, providing a detailed understanding of how injury epidemiology interacts with season and culture. By separating admission frequency from injury severity, this study reveals a key epidemiologic feature that aligns with findings from other studies in high-income and low-and middle-income settings. While burn admissions remain statistically consistent throughout the year, injury severity changes dramatically by season. Specifically, despite stable admission counts, burn severity measured by total body surface area (TBSA) increased during the winter months, revealing a clear dissociation between frequency and severity that mirrors winter's severity peaks, which was reported in other studies from Portugal, Turkey, and Switzerland, where colder months are associated with greater TBSA and deeper injuries [3-5]. Our finding underscores that seasonality can manifest as either frequency-driven or severity-driven, depending on local exposure contexts.

The stability of overall admission rates is a novel observation for this region and is explained by the consistent pediatric baseline: pediatric admissions (<15 years) were evenly distributed across seasons and showed no seasonal influence on regression analysis, reflecting a continuous domestic risk that persists regardless of environmental conditions [21]. This pediatric “steady state” has been documented in the WHO Global Burn Registry, where children aged 1-5 years constitute 62% of pediatric entries and exhibit scald predominance year-round [21]. Adult admissions, in contrast, displayed predictable seasonal fluctuations, with a clear peak in spring and a nadir in summertime, which is a pattern reminiscent of the spring peak observed in German and Dutch burn unit data, where longer daylight hours and increased outdoor work correlate with higher flame burn incidence [4]. However, these adult‑specific differences did not achieve statistical significance after adjustment for exposure time and were insufficient to alter aggregate admission patterns because the stabilizing effect of pediatric cases buffered overall volume. This explains why the visually apparent adult seasonality did not persist in the final regression models and highlights how pediatric epidemiology can mask adult temporal trends when analyzed in aggregate.

The most clinically significant finding of the study is the increase in burn intensity during the winter. The median TBSA in winter was five times higher than in summer (p = 0.012), indicating a significant seasonal increase in injury severity despite a steady admission volume, which is in alignment with Turkish and Iranian studies reporting similar TBSA outcomes across seasons [4,16]. Similarly, the probability of severe burns (≥20% TBSA) was numerically higher in winter, but not statistically significant. Taken together, these patterns suggest a seasonal shift in exposure rather than a change in burn mechanisms. In practical terms, winter creates several risk conditions at home, including extended contact with hot water, higher tap-water temperatures from heating systems, and increased use of heaters, all of which can exacerbate scald severity while remaining coded under the same mechanism. Furthermore, cultural habits in the region, particularly the preparation and consumption of hot tea and Arabic coffee around open fires during winter gatherings, increase the risk of accidental spills and contact injuries. As a group, these factors offer a more plausible explanation for the rise in burn intensity during winter than mechanism distribution alone.

Sex-stratified analysis provides further context for the observed winter increase in burn severity. Although winter admissions represented a higher proportion of annual cases among females than males, injury severity did not differ significantly between genders. This pattern contrasts with reports from the UAE and Southwestern Saudi Arabia, where male predominance in burn admissions has been linked to workplace-related exposures [17,19]. In our cohort, the absence of a sex-based difference in TBSA suggests that occupational exposure is unlikely to be the primary driver of winter-associated burn severity. Instead, the findings point toward shared household risk factors affecting both sexes. Winter-related domestic exposures, including elevated hot-water temperatures from household heating systems, prolonged bathing during colder months, and contact with heating-related devices, including hot-water bags or warming pads, may increase burn extent while remaining classified under broad mechanism categories such as scalds. This interpretation is consistent with evidence from an East Mediterranean systematic review, which identified the home as the injury location in 72-94% of burn cases and highlighted indoor heating practices as major contributors to winter burns [7].

Cultural periods revealed distinct but circumscribed temporal clustering. Ramadan accounted for 8.1% of admissions, a proportion consistent with its duration in the lunar calendar, suggesting no aggregate surge in burn volume. Although the distribution of burn mechanisms during Ramadan did not differ significantly from that in non-Ramadan periods, the observed predominance of scald and flame injuries suggests a predictable risk window associated with intensified evening meal preparation, echoing trends across the Middle East where holiday-related cooking spikes correlate with pediatric injuries [2,7]. Similarly, while Eid al-Fitr and Eid al-Adha were associated with identifiable admission clusters, these did not constitute statistically significant elevations in daily rates. Rather, their temporal concentration reflects predictable exposure patterns linked to holiday-specific activities. Consequently, these findings advocate for targeted, event-specific prevention messaging over broad seasonal interventions, supporting the established efficacy of culturally tailored education [19].

This study has limitations due to its retrospective, single-center methodology. Although King Fahad Specialist Hospital serves as the regional referral center, small burns treated in primary care or private clinics may be underrepresented, thereby biasing the cohort towards more serious injuries. Burn mechanisms were defined using broad clinical categories, which may have obscured contextual distinctions within mechanisms, particularly for scald injuries. Detailed information on water temperature, duration of exposure, heating device type, and prehospital delay was unavailable, making it impossible to draw definitive causal conclusions about winter harshness. Furthermore, socioeconomic indicators were not collected, limiting the estimation of household vulnerability. Future prospective or mixed-methods investigations should incorporate these characteristics.

## Conclusions

This seven-year regional analysis elucidates a distinct dissociation between seasonal burn frequency and burn extent (TBSA %) in the Qassim region. While admission volume remained statistically stable year-round, underpinned by a persistent and consistent pediatric baseline, TBSA % increased significantly during the winter months. This seasonal escalation in TBSA % was independent of shifts in mechanism distribution, flame-specific severity, or sex, highlighting the decisive impact of winter-associated domestic exposures. These data support a bifurcated prevention strategy: pediatric burn prevention must remain a continuous, year-round domestic priority, while population-level interventions should be intensified during winter to mitigate injury severity through targeted education on domestic hot-water safety and heating device regulation. Ultimately, incorporating a focus on severity mitigation alongside traditional frequency-reduction efforts allows for a more precise public health response, optimizing the allocation of specialized burn resources and enhancing overall patient outcomes.

## Appendices

**Table 5:** Supplementary table 1-STROBE checklist for observational studies Adapted from the STROBE (Strengthening the Reporting of Observational Studies in Epidemiology) Statement [20].

Item No.	STROBE Recommendation	Location in the Manuscript
Title & Abstract		
1(a)	Indicate study design in title or abstract	Methods: “retrospective registry analysis”
1(b)	Provide an informative and balanced abstract	Abstract: Background, Methods, Results, Conclusion
Introduction		
2	Explain scientific background and rationale	Introduction: global burden, seasonality, regional gap
3	State specific objectives	End of Introduction: Aim paragraph
Methods		
4	Present key elements of study design early	Methods 2.1: Retrospective observational analysis
5	Describe setting, locations, and dates	Methods 2.1–2.2: KFSH, Qassim; Nov 2018–Nov 2025
6(a)	Eligibility criteria and participant selection	Methods 2.2: Inclusion/exclusion criteria
6(b)	Methods of follow-up	Not applicable - registry-based retrospective study
7	Clearly define outcomes, exposures, predictors	Methods 2.3: Seasons, Ramadan/Eid, TBSA, age groups
8	Data sources and measurement methods	Methods 2.3–2.4: Hospital registry, TBSA assessment
9	Address potential sources of bias	Discussion - Limitations: Single-center, referral bias
10	Explain study size	Methods 2.2: All eligible admissions (n=816)
11	Explain handling of quantitative variables	Methods 2.5: TBSA medians/IQR; age stratification
12(a)	Statistical methods used	Methods 2.5: Chi-square, Kruskal-Wallis, NB regression
12(b)	Methods for subgroup analyses	Methods 2.5.1 & 2.5.2: Age- and sex-stratified analyses
12(c)	Handling of missing data	Methods 2.4: Complete-case analysis; TBSA 98.8% available
12(d)	Address loss to follow-up	Not applicable
12(e)	Sensitivity analyses	Not performed
RESULTS		
13(a)	Numbers at each stage of study	Results 3.1: Total cohort n=816
13(b)	Reasons for non-participation	Methods 2.2: Defined exclusions
13(c)	Flow diagram	Not included (single-stage registry cohort)
14(a)	Characteristics of study participants	Results 3.1 & Table 1
14(b)	Missing data for variables	Methods 2.4: Reported
15	Outcome data	Results 3.2–3.6; Tables 2–4; Figures 1–7
16(a)	Main results with estimates	Results 3.2–3.6: IRRs, TBSA medians
16(b)	Category boundaries reported	Methods 2.3: Pediatric <15 years; TBSA groups
16(c)	Translate estimates to clinical relevance	Discussion: Severity vs frequency interpretation
17	Other analyses	Results 3.3–3.6: Age, sex, cultural periods
Discussion		
18	Summarize key results	Discussion – Opening paragraphs
19	Discuss limitations	Discussion – Limitations section
20	Provide cautious interpretation	Discussion – Comparative analysis
21	Discuss generalisability	Discussion – Regional referral context
Other Information		
22	Funding	This study received no external funding.

**Table 6:** Supplementary table 2-Ramadan time windows

Ramadan periods	Start	End
1	2019-05-06	2019-06-03
2	2020-04-24	2020-05-23
3	2021-04-13	2021-05-12
4	2022-04-02	2022-05-01
5	2023-03-23	2023-04-21
6	2024-03-11	2024-04-09
7	2025-03-01	2025-03-30

**Table 7:** Supplementary table 3-Eid al-Adha time windows

Eid adha period	start	end
1	2019-08-11	2019-08-14
2	2020-07-31	2020-08-03
3	2021-07-20	2021-07-23
4	2022-07-09	2022-07-12
5	2023-06-28	2023-07-01
6	2024-06-16	2024-06-19
7	2025-06-06	2025-06-09

**Table 8:** Supplementary table 4-Eid al-Fitr time windows

Eid alfitr periods	start	end
1	2019-06-04	2019-06-06
2	2020-05-24	2020-05-26
3	2021-05-13	2021-05-15
4	2022-05-02	2022-05-04
5	2023-04-22	2023-04-24
6	2024-04-10	2024-04-12
7	2025-03-31	2025-04-02

**Table 9:** Supplementary table 5-seasonal distribution of burn admissions stratified by age group

	Age group	Season	Number of cases	% of the cases
1	Adult (≥15y)	Autumn	126	25.5
2	Adult (≥15y)	Spring	141	28.5
3	Adult (≥15y)	Summer	113	22.9
4	Adult (≥15y)	Winter	114	23.1
5	Pediatric (<15y)	Autumn	79	24.5
6	Pediatric (<15y)	Spring	79	24.5
7	Pediatric (<15y)	Summer	79	24.5
8	Pediatric (<15y)	Winter	85	26.4
